# Transdermal Delivery Systems of Natural Products Applied to Skin Therapy and Care

**DOI:** 10.3390/molecules25215051

**Published:** 2020-10-30

**Authors:** Ying-Chen Cheng, Tzong Shiun Li, Hong Lin Su, Po Chun Lee, Hui-Min David Wang

**Affiliations:** 1 Graduate Institute of Biomedical Engineering, National Chung Hsing University, Taichung City 402, Taiwan; wizozd51221@gmail.com; 2 Department of Plastic Surgery, Show Chwan Memorial Hospital, Changhua 500, Taiwan; li.tsa2@icloud.com; 3 Department of Life Sciences, National Chung Hsing University, Taichung City 402, Taiwan; suhonglin@nchu.edu.tw; 4 Cardiovascular Clinic, Kaohsiung Armed Forces General Hospital, Kaohsiung City 802, Taiwan; 5 Graduate Institute of Medicine, College of Medicine, Kaohsiung Medical University, Kaohsiung City 807, Taiwan; 6 Department of Medical Laboratory Science and Biotechnology, China Medical University, Taichung City 404, Taiwan

**Keywords:** natural products, skin, transdermal delivery, liposome, emulsion, lipid nanoparticle

## Abstract

Natural products are favored because of their non-toxicity, low irritants, and market reacceptance. We collected examples, according to ancient wisdom, of natural products to be applied in transdermal delivery. A transdermal delivery system, including different types of agents, such as ointments, patches, and gels, has long been used for skin concerns. In recent years, many novel transdermal applications, such as nanoemulsions, liposomes, lipid nanoparticles, and microneedles, have been reported. Nanosized drug delivery systems are widely applied in natural product deliveries. Nanosized materials notably enhance bioavailability and solubility, and are reported to improve the transdermal permeation of many substances compared with conventional topical formulations. Natural products have been made into nanosized biomaterials in order to enhance the penetration effect. Before introducing the novel transdermal applications of natural products, we present traditional methods within this article. The descriptions of novel transdermal applications are classified into three parts: liposomes, emulsions, and lipid nanoparticles. Each section describes cases that are related to promising natural product transdermal use. Finally, we summarize the outcomes of various studies on novel transdermal agents applied to skin treatments.

## 1. Introduction

A natural product is a chemical compound or ingredients produced by a living organism. In the broadest sense, natural products include any substance yielded by life. Natural product application has more than 3000 years of history and includes many bioactive phytochemicals derived from plants and animals. Moreover, natural products have been recorded in many books in ancient China. The earliest is the “Wǔshí’èr bìng fāng” unearthed from Ma Wangdui. The first medical book in China, “Huángdì nèijīng·sù wèn”, had a discussion on the production and clinical application of plasters. In the Ming Dynasty, the “Běncǎo gāngmù Compendium of Materia Medica” recorded the application of topical plasters to treat gangrene and rheumatism. In the Qing Dynasty, topical plasters had become popular medicine. “Jí guǎng shēng jí” and “Lǐ lún piánwén” are two monographs in the history of China dealing with external treatments, clarifying the principles of external treatment and internal treatment. Many diseases are treated through the skin route. Our predecessors have long recognized some theoretical problems in the external application of herbal medicines plasters, such as acupoint application and meridian therapy. These theories have played a guiding role in the treatment of internal and external diseases of Chinese medical. Acupoint application of Chinese medicines often use a small dose of the medicine to achieve the effect compared to a larger oral dose [1]. Concerning the natural product application method, skin contact is with all of the body, including the heart, liver, spleen, lung and kidney; therefore, the ingredients penetrate each part. Placing natural products on the acupoint can treat illness, and exert some special efficacies. At present, transdermal natural products still have a mainly local effect [2]. Regular dosages are highly concentrated, and new technologies for prescription drugs are needed to overcome the skin barrier, especially the stratum corneum barrier, to improve the local consequence or systemic effects.

**Figure 1:**
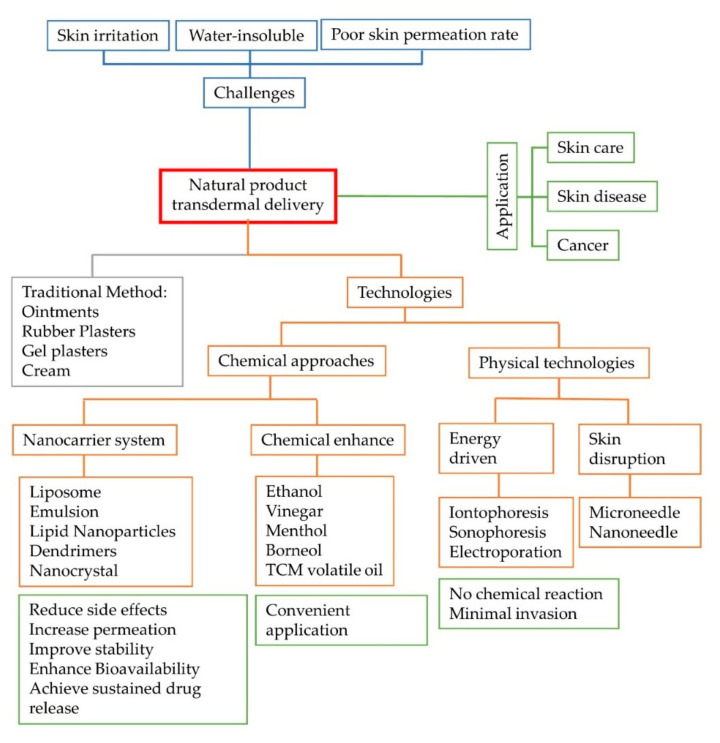
The applications, challenges, and technologies of natural product delivery on human skin.

The meridian penetration point in the transdermal drug system is based on the herbaceous medicine meridian principle. Agents accumulate through a meridian point to reach a higher concentration than those administered by the injection or oral form. Research shows that agents absorbed by the meridian point principle are different from those absorbed by blood vessels, because a drug penetrates by a meridian point, it treats the lesions directly and does not circulate through the whole body. In previous studies on herbal medicines absorbed by a meridian point, the medicines were observed to go through the meridian—not dispersed and spread on other locations [3]. This application enhances the treatment efficiency and reduces the dose absorbed by the circulatory system to avoid having huge drug amounts entering the body, potentially causing toxicity and side-effects by long-term medication. Regarding the mechanism of the transdermal drug system by meridian points, it can be introduced from several aspects. In recent years, meridian biophysics demonstrate that the paths have low impedance and specific acoustic characteristics and corresponding thermal change [4]. In the meridian transduction process, mast cells release several kinds of bioactive materials to elevate the excitation of nerve endings in the skin’s outer layer, resulting in a sensitivity effect; therefore, the mast cell is called an activator. The effect involves the cooperation of the nerves, hormones, and immune system. Hormones, receptors, cyclases, cyclic adenosine monophosphate (cAMP), and protein kinases are an amplification system within an organism, and even a very small stimulus can produce effects approximately more than ten thousand times greater. The patch contains Chinese medicines attached to a meridian point, and the bioactive components, such as *Typhonium giganteum Engl.* [5], *Evodia rutaecarpa (Juss.) Benth.* [6], and *Aconitum carmichaelii Debx*. [7] affect cell receptors, for example, activation of the β-adrenergic receptor. Although they contain extremely low concentrations, their effects are made possible through the abovementioned physiological amplification. The meridian system is a multilevel, multifunctional, and multiform three-dimensional regulatory system. It is believed that the meridian point transdermal administration route will open a new therapeutic prospect for the treatment of tumors and other chronic diseases [8]. Nowadays, many novel transdermal applications have been reported. In this review, we summarized and discussed the outcomes of different studies on novel herbal medicines applied on the skin (Figure 1).

## 2. Natural Products in Transdermal Drugs

The percutaneous absorption of a drug is a complicated process; the penetration rate is influenced by the molecular size, hydrophile/lipophile balance, melting point, and water solubility. A natural product compound is mostly applied in the clinical setting. In general, transdermal natural product drugs are composed of drugs that activate blood circulation (borneol, lilac, pepper [9]), and strong irritants (white mustard, ginger onion, garlic, leek). These natural agents have a long history of transdermal administration, and Table 1 shows an example that has been researched [2,10]. In ancient society, due to the limitations of social healthy conditions, medicine powders or crude herbal extracts were applied in formulations, such as topical powders, pastes, and pills. The dosage form for skin administration is a powder mixed with water, honey, wine, or vinegar, but the disadvantages are: poor adhesion and being messy; it may have a high bacterial content and easily cause skin infection; it is difficult to preserve and simply develops mold; the percutaneous penetration of the drug components is low, etc. Industrial production of many traditional dosage forms is difficult, or the quality is hard to control, which limits application in modern clinical practice. However, simple acquirement and convenient usage make Chinese medicines still widely utilized in some clinical cases. Currently, natural product transdermal preparations are commonly used in the form of ointments, patches, and gels.

### 2.1. Ointments

The ointment is made by Zhong Yao Yin Pian (prepared herbal medicine in small pieces for decoction), vegetable cooking oil with red lead or cerussite, and spread on the mounting materials to produce a topical medication. Ointment that is produced with red lead is called black ointment, and ointment that is made with cerussite is called white ointment. Ointments can be used for disinfection, detoxification, myogenesis, removal of wind-cold, removal of rheumatism, and activation of the blood. Currently, black ointments are usually used in the clinic, and their production process involves deep-frying in edible vegetable oil followed by filtration and interaction with yellow lead. The advantages are the treatment effect, hot pack effect, and higher adhesion, but the active compound is easily lost since it is produced at high temperature. Before using it, it must be heated to soften the ointment. Because the ointments contain red lead, they must be detoxified. Without detoxification, it will cause skin toxicity [44].

### 2.2. Rubber Plasters

Rubber plasters are made from rubber, resin, fat, lipophilic excipients, and the drug. All of the ingredients are mixed and spread onto the mounting materials to produce a topical medication. Because natural rubber and natural rosin may cause irritation, synthetic resin and rosin derivatives have replaced these natural ingredients to reduce the irritation. The drug load in the rubber plaster is relatively low because the water content influences the adhesion, and the rubber pulp cannot take up the large therapeutic medicine molecules. In recent years, a hot-melt adhesive has replaced rubber plasters since it does not include adhesion gel or filler and it is associated with less irritation [45].

### 2.3. Gel Plasters

Gel plasters are drugs mixed with hydrophilic matrix and spread on mounting materials. This preparation has many advantages, such as ease of use, wide dosage range, proper adhesion, increased humidity, great bioavailability, and a production process without the need for toxic solvents [46]. Furthermore, gel plasters enhance the stratum corneum hydration to increase the penetration rate. These are more suitable than rubber plasters for water-soluble and lipid-soluble drugs because gel plasters can absorb a higher dosage. This corresponds to the property of higher dosage ingredients and a complex compound. However, gel plasters can be easily removed since the gel dehydrates, and its hydrophilic matrix promotes lipid-soluble monomer release to further decrease the therapeutic effect [47].

## 3. Novel Applications in Natural Product Transdermal Delivery

**Figure 2:**
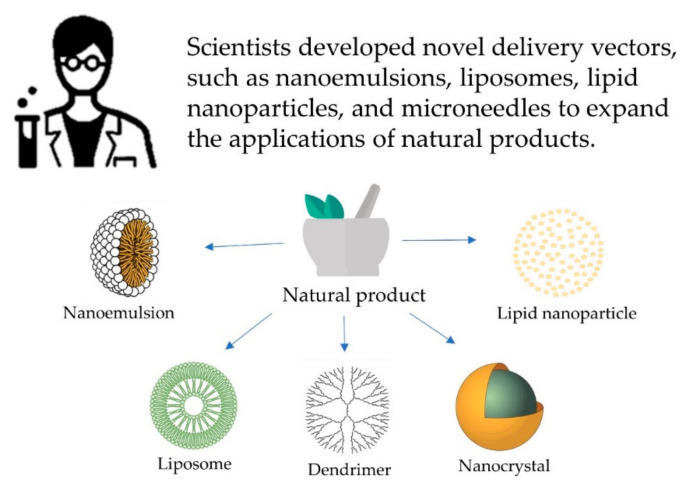
Scientists developed novel vector to expand the application of natural products.

Scientists developed novel delivery vectors, such as nanoemulsions, liposomes, lipid nanoparticles, and microneedles to expand the applications of natural products (Figure 2).

### 3.1. Nanocarrier

In recent years, people have focused on the modernization of natural product as an important topic. Production and experimentation have greatly developed and advanced. In particular, new separation technology was widely applied in natural medicine, such as supercritical fluid extraction, ultrasonic extraction and microwave-assisted extraction. High purity is achieved by the extraction approaches developed [48], and realize natural product nanosized. Designed several formulations for special disease and improving the ability of target and effective. Solubility and bioavailability are biggest issue in the drug industry. The drug can be stable and shrunken by the nanotechnology to improve stability and solubility, and to enhance the bioavailability through the surface area increase contacting to the gastrointestinal tract. Nanosized drug delivery systems notably enhance the bioavailability and solubility of active ingredients by penetrating vital cellular stocks. The nanosized drug was being reported to improve the transdermal permeation of many drugs when compared to conventional topical formulations [49]. Therefore, natural product drugs have been made into nanosized drugs to improve the penetration effect.

### 3.2. Liposome

In the early 1980s, some research introduced liposomes as skin drug delivery systems; they were initially developed primarily for localized effects with minimal systemic delivery. Liposomes are phospholipid vesicles including one or more bilayers encircling an aqueous core. The liposomal formulations effectiveness has been manifested in transdermal drugs, topical drugs, hair follicles, etc. for molecules ranging from low molecular weight drugs to proteins and peptides [50]. To date, many studies have combined conventional liposomes with topical drug delivery. However, there is no understanding of the mechanism by which liposomes enhance drug penetration. The Cevc G’s team declared that Transfersomes^®^ not only penetrate the deeper layers of the skin, but also enter the systemic circulation through topical application. Other research articles suggest that liposomes enhance drug penetration by fusing with the stratum corneum lipids or disturbing the intercorneocyte lipid composition of the penetration-enhancing mechanism [51]. Applying a liposome as a transdermal delivery method could improve the penetration rate and overcome the skin barrier. Its therapeutic effect for the deep skin and subcutaneous tissue is better, and it allows continuous treatment.

Resveratrol (3,4′,5-trihydroxystilbene, RSV) is isolated from the roots of *Polygonum cuspidatum* [52]. The transdermal delivery of RSV is an attractive option compared with other administration routes in order to avoid gastrointestinal problems and first-pass metabolism. However, the therapeutic obstacle for the RSV transdermal delivery is its poor permeability. In addition, the solubility of RSV is low (0.03 g/L), and its stability in water is deficient due to photoisomerization that reduces its activity [53]. Pey-Shiuan Wu et al. used a novel type of carrier, a transfersome, to load RSV. A transfersome is produced with phosphatidylcholine and nonionic edge activators. It can increase the lipid bimolecular membrane of transfersome flexibility and allow drugs to penetrate the skin owing to its ultradeformability [54]. Donato et al. developed ultradeformable liposomes loaded with resveratrol and 5-fluorouracil to evaluate the potential treatment of nonmelanoma skin cancer. They found that the liposomes could modify the 5-fluorouracil effect and enhance the resveratrol activity. Ultradeformable liposomes may gather in deeper skin layers, therefore, generating a cutaneous storehouse from which resveratrol and 5-fluorouracil are slowly released [55].

Danshensu, also called danshencarboxylic acid, is the main water-soluble phenolic acid component in *Salvia miltiorrhiza*. It is mainly used for the treatment of cardiovascular diseases. Yang-De Zhang et al. developed a danshensu liposome transdermal drug delivery system. The prepared ultradeformable nanoliposomes had double-layer sphericity, and the average encapsulation percentage was 45.8%. This system has the potential for cardiovascular disease treatment [56]. Paclitaxel has been shown to have outstanding anticancer activity in both first- and second-line settings for breast cancer [57,58]. Paclitaxel is insoluble in water and has several side-effects that limit its application in the clinic. At present, the clinical parenteral dosage form of paclitaxel is dissolved in a 1:1 mixture of cremophor EL (poly-oxyethylated castor oil):ethanol. However, cremophor may have toxicity and produces vasodilation, labored breathing, hypersensitivity, cardiotoxicity, nephrotoxicity, and neurotoxicity [59]. Therefore, many researchers have attempted to develop a cremophor-free paclitaxel formula. Puneet et al. developed an elastic liposome-based paclitaxel dosage form that did not contain cremophor EL. The elastic liposomal formulation loaded the maximum amount of paclitaxel and reached 6.0 mg/mL. The hemolytic toxicity assay revealed that the elastic liposomal formulation had less toxicity than the commercial products [60]. Many studies have focused on the application of the liposome as a carrier for natural product because of its advantages, such as relieving adverse effects, improving the localization effect, avoiding gastrointestinal problems and first-pass metabolism and avoiding natural product degradation.

### 3.3. Emulsions

Emulsions are made up of two immiscible liquid phases by using mechanical shear and surfactant. According to the surface-tension theory of emulsification, the emulsifiers or surfactants reduce the interfacial tension between the two immiscible liquids, lowering the repellent force between the two immiscible liquids and decreasing the attraction between the molecules of the same phase. The choice of surfactant is according to the hydrophilic-lipophilic balance (HLB) value or critical packing parameter (CPP) helping to develop the desired emulsion [61,62], and strictly limiting its clinical application. Transdermal delivery is a better route for TPL than oral administration since it prevents the first-pass metabolism in the liver and reduces damage to the gastrointestinal tract, liver, and kidney [63]. Meng Yang et al. created TPL nanoemulsion gels. In vitro, the TPL nanoemulsion gels and TPL nanoemulsions had greater cumulative amounts penetrate rat skin than the TPL nanoemulsion gels [64].

Paeonol is the active compound of cortex moutan. It has a low melting point, high volatility, and poor water solubility. Paeonol has poor oral bioavailability owing to its rapid and complete first-pass metabolism; thus, it is absorbed rapidly after oral administration. Maofu Luo et al. developed microemulsion gel formulations that had a higher penetration rate in excised rat skin. Compared with orally administered paeonol suspension, the microemulsion gel enhanced the bioavailability by 1.28-fold [65]. Evodiae fructus has pharmacological effects such as cardioprotective, anti-arrhythmic, anti-inflammatory and analgesic effects. Zhang Guang-Chang et al. developed a cataplasm loaded with an Evodiae fructus microemulsion. The cumulative permeation quantity of the Evodiae fructus oil-in-water microemulsion cataplasm was 1.86-fold higher than that of the conventional cataplasm. This indicates that the oil-in-water microemulsion cataplasm can improve the transmittance of herbal medicine fat-soluble components [66]. Pharmacological experiments have confirmed that bulleyaconitine A (Bu-A) has obvious analgesic and anti-inflammatory effects. However, the margin of safety is relatively narrow. When administered orally or by injection, the fluctuations in blood concentration and side effects are substantial [67]. Xiaohui, Wu prepared the water-insoluble drug Bu-A as an oil-in-water microemulsion with a size of 100 nm to improve the solubility and chemical stability and promote transdermal absorption. The biological absorption is 3–4 times higher than that of the adhesive-bonded water insoluble matrix patch [68]. Encapsulating cumin essential oil (CEO) in transdermal nanoemulsion formulations to acquire efficient and prolonged systemic antioxidant and hepatoprotective activities [69]. *Clinacanthus nutans Lindau* (C. nutans) is a well-known medicinal plant in South-East Asia but it is hard to deliver. A nanoemulsion has been chosen to be a carrier in the encapsulation of bioactive ingredients of C. nutans extract to improve its solubility and bioavailability [70].

### 3.4. Lipid Nanoparticles

Lipid nanoparticles are composed of a solid lipophilic matrix and active molecules that can be entrapped. The distribution range of the particle size is between 150–300 nm. The smaller sizes, e.g., <100 nm or larger sizes to up to 1000 nm can be obtained for special needs [71]. In the 1990s, scientists developed the ‘solid lipid nanoparticles’ in order to combine the advantages of solid particles, emulsions, and liposomes. SLNs were made by exchanging the liquid lipid (oil) of the emulsions with a solid lipid, i.e., a lipid that is solid at room temperature and body temperature. In recent years, SLNs have gradually begun to be used in topical preparations. The other type of lipid nanoparticle is nanostructured lipid carriers, which are improved SLNs. NLCs also consist of a mixture of solid and liquid lipids, but with the solid lipid in a greater amount [72].

Yongwei Gu et al. developed nanocarriers loaded withTPL. Compared with the control treatment, TPL nanoparticles could disorder skin structure, enhance keratin enthalpy and decrease the SC infrared absorption peak area. In addition to their great anti-inflammatory effects, TPL nanoparticles, NLCs, and SLNs could serve as promising nontoxic agents for transdermal drug delivery systems [73]. Vincristine (VCR) is a dimer-indo-alkaloid that was extracted from the leaves of *Catharanthus roseus*. VCR is most often used to treat acute lymphocytic cell leukemia, Hodgkin and non-Hodgkin lymphoma, breast cancer [74], neuroblastoma, and so on. When administered intravenously, VCR is rapidly widely dispersed and reaches a high concentration in nerves and muscles [75]. VCR causes a neurotoxicity, though constant intravenous injection avoids causing toxicity by high drug concentrations, it elongates the time the drug stays in the blood. However, this treatment causes many issues with patient compliance. Therefore, Lu Yan developed dry-film and ultrasonic dispersion methods, namely, producing vincristine-loaded transfersomes that were prepared with Brij 78 as a surfactant and chitosan as a carrier ingredient by the ionic gelation of chitosan with TPP [76,77]. VCRs could penetrate the skin and be targeted delivered to lymph. Transdermal drug delivery can improve the therapeutic effect of VCR and reduce its adverse effects. Lutein is a natural carotenoid and a member of the xanthophyll family. It can quench singlet oxygen, a highly reactive free radical that can damage DNA [78]. Similar to other antioxidants, lutein is hydrophobic and unstable since it is an isoprenoid polymer that contains many conjugated double bonds that can be easily isomerized, oxidized and degraded. Due to its insolubility in water, it has moderate bioavailability [79]. To solve these problems and to increase its stability, lutein can be entrapped in nanocarrier. Khalil Mitri et al. developed lutein loaded into nanocarriers. The nanocarriers showed a perfect ability to protect lutein from UV degradation [80]. Capsaicin is an alkaloid extracted from chili peppers that is widely used to treat pain and inflammatory diseases in clinical settings [81]. However, capsaicin always causes an itching, pricking, or burning sensation [82]. Therefore, Xia-Rong Wang et al. designed effective and safer carriers for the capsaicin application, namely, capsaicin-loaded lipoidal nanocarriers. These exhibited sustained release and safe properties. They can also significantly increase the penetration amount, permeation flux, and skin retention of capsaicin because of the nanocarriers. Studies have shown that the metabolism in human skin is slower and has a longer elimination half-life in the skin applications when delivered via nanocarrier [83]. Salidroside reduces the proliferation of melanocytes, whereas paeonol downregulates tyrosinase activity in melanocytes; the ideal delivery method to prevent pigmentation via multiple pathways is the fast release of salidroside followed by the continued release of paeonol [84]. To avoid drug degradation and to decrease their cytotoxicity at high dosages, a novel nanocarrier system to deliver salidroside and paeonol was constructed [85,86]. A novel nanocarrier-gel for sequential delivery of salidroside and paeonol was developed. In UVB-stimulated guinea pig skin [87], a nanocarrier-hydrogel system loaded with salidroside and paeonol was more able to decrease melanin levels than the other treatments. The nanocarrier-hydrogel system has great potential in nanomedicine [88].

### 3.5. Others

In addition, dendrimers and crystals are also applied for natural product transdermal delivery. Compare with liposomes, nanoemulsions, and lipid nanoparticles, these methods are not used as commonly in natural product delivery; thus, we will briefly introduce the two methods.

Dendrimers were first discovered by Fritz Vogtle in 1978. Dendrimers are nanosized, radially symmetric molecules that are homogeneous and nearly monodisperse, consisting of symmetric branching units constructed with a small molecule or a linear polymer core [89]. Therefore, dendrimers can be engineered to suit a particular application for entrapment of small molecules and biologics, and they can be used for all routes of administration. They can be applied in anticancer drugs, transdermal drug delivery [90,91], gene delivery, enhancing solubility, and photodynamic therapy.

Photochemotherapy is a necessary treatment for a variety of skin diseases. Among these therapies, the most used is psoralens and long-wavelength ultraviolet radiation (PUVA) with psoralens administered orally because systemic PUVA therapy very effective and simple to execute [92,93]. The frequently applied psoralens are 8-methoxypsoralen (8-MOP), but the 8-MOP oral administration causes gastrointestinal side-effects and elevates the risk of serious complications such as carcinogenesis or glaucoma. In the study by Borowska et al., 8-MOP complex loaded in G3 and G4 polyamidoamine dendrimers enhanced the permeation rate of the 8-MOP in the deeper layers of the skin and led to higher concentrations as shown by confocal laser scanning microscopy. Therefore, this novel transdermal drug delivery system improved its permeation and achieved of skin disease with PUVA therapy more safely and effectively [94,95].

Drug nanocrystals are nanometer-size crystals, which imply they are nanoparticles with a crystalline property. A further property is that nanocrystals consist of 100% drug. Nanocrystals increase the dissolution velocity by surface area expansion to enhance the bioavailability and the saturation solubility since the concentration gradient between the gut lumen and blood is elevated; thus, the absorption occurs by passive diffusion. Dispersion of drug nanocrystals in liquid media leads to so-called “nanosuspensions” (in contrast to “microsuspensions” or “macrosuspensions”). To stabilize the dispersed particles, surfactants or polymeric stabilizers are added, and the dispersion media can be water, aqueous solution or nonaqueous phase [96]. Nanosuspensions have some advantages, such as high entrapment efficiency, low induction of side effects by the excipients, and comparatively low cost.

Berberine can inhibit gram-positive and gram-negative bacterial growth. Moreover, poly(N-Isopropylacrylamide) is an intelligent hydrogel that has a lower critical solution temperature of approximately 32–34 °C [97,98], and it is suitable for wound repair [99]. Thus, He Xu et al. combined two properties to develop a novel berberine nanosuspension loaded in a hydrogel-grafted fabric. It has many properties that facilitate the repair of infected skin wounds, such as sustained drug release dynamics, excellent water absorption, and easy dressing removal after use [100,101].

All things considered, nanocarriers overcome the problem of natural product transdermal drugs by improving drug solubility and stability, achieving sustained drug release, increasing permeation and bioavailability, avoiding gastrointestinal problems and first-pass metabolism, decreasing skin irritation, eliminating the use of toxic solvents and allowing sequential delivery. Recent examples of using nanocarriers for natural product delivery are listed in Table 2.

## 4. Skin Care Applications

In ancient times, people used *Impatiens balsamina Linn*. to decorate the nails, applied natural indigo to draw the eyebrows, and used oil from animals to moisturize the skin. These are the original cosmetics made by herbal medicine. In related ancient books, Yang Kui Fei applied tài zhēn hóng yù gāo; Wu Zetian used tiān hòuyì mǔ cǎo miàn fang to remove spots; and the Empress Dowager used jiāyù róngsàn to care for her skin. According to Chinese medical books, the “Wǔshí’èr bìng fang” in the Warring States period recorded external application of Chinese medicine to remove warts and scars. Traditional eastern physicians paid attention to the cosmetics; Shennong Bencaojing, Taiping Shenghui Fang, and Compendium of Materia Medica include related records. The first book about Chinese medicine cosmetics is zhuāng tái fāng, which was published in the Sui Tang Dynasty. The pǔjìfāng was published in the Ming Dynasty and is currently known as the Chinese beauty collection. The book collected previous skin care formulas and promoted the development of traditional Chinese medicine cosmetics [112].

The development of natural product cosmetics has had some deficiencies and difficulties. First, cosmetics are made according to Chinese medical principles and are not simply extracts of natural product added into cosmetics. Second, although they are safer than chemical ingredients, safety is essential. Natural product content is affected by the environment, and it may contain heavy metals or other factors. Some natural products have a narrow range of safe doses [113]. Natural product cosmetics can easily be contaminated, produce toxicity, or lose their effects, and this should also be considered. Careful quality analysis and clean production processes are essential. Third, formulation is difficult because extracts of natural product have ions, acids, and mucopolysaccharides that would break down lotions and creams. The developer should consider the most appropriate cosmetic formula and ingredients. Finally, there are no uniform quality control standards for natural product cosmetics, and this is an issue that should be resolved [114].

**Figure 3:**
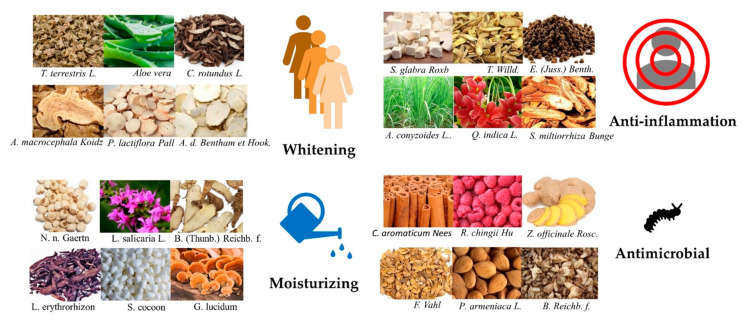
Natural products in various types of therapeutic bio-functions.

In the early years, the active ingredients for skin whitening included mercury compounds, hormone-like compounds, and aluminum hydroxide; their effects are rapid and significant, but these compounds are easily deposited in the body and are highly toxic and irritating. Currently, widely used whitening agents, such as kojic acid and vitamin C derivatives also have potential safety hazards, poor stability, or slow efficacy [115,116]. The natural product active ingredient as a whitening agent has the advantages of safety, mildness, long-lasting, high efficiency, etc. Because of their low toxicity and side effects and high safety and cost effectiveness, natural product whitening products are favored by many people. These products improve skin complexion by promoting blood circulation, reducing melanin content, providing antioxidant protection, and inhibiting melanocyte proliferation [117,118]. At present, most whitening cosmetics, such as a variety of flavonoids, polyphenols, floral acid, and other active ingredients of natural product, target tyrosinase. Currently, researchers are focused on applying natural products in cosmetics. In Table 3 and Figure 3, we listed some natural products that could be applied in cosmetics [119].

## 5. Conclusions

Novel transdermal methods facilitate natural product development, enhancing the natural product transdermal permeation rate. However, most research on transdermal delivery methods has only focused on monomers, but some herbal medicines are complex ingredients that interact with different compounds to achieve therapeutic effects. Furthermore, natural products in traditional methods use high dosages, while nanotechnology can only carry low dosages; thus, it is difficult to effectively load natural product in nanocarriers. For these reasons, it is important to research how to apply novel nanocarriers to achieve an authentic natural product effect. Another issue is that most investigations have studied effectiveness, but did not further study the systemic mechanism. In this regard, there are many important problems needed to be solved, such as creating a suitable transdermal method and developing proper assessments for natural products. To sum up, we concluded these filed published works within the present article.

## Figures and Tables

**Table 1:** Examples of transdermal natural product agents.

The Effect of Transdermal Drug	Natural Product Active Ingredients
Analgesic and anti-inflammation	Capsaicin [11], aconitine [12], turpentine [13], strychnine [14], triptolide (TPL) [15], sinomenine [16], colchicine [17], curcumin [18], berberine [19], lycopene [20], glycyrrhetic acid [21], catechin [22], geniposide [23], resveratrol [24], andrographolide [25], paeonol [26], mangiferin [27],
Skin repair	Camptothecin derivatives [28], Astragaloside IV [29], Asiaticoside [30]
Antitumor	Bufalin [31], podophyllotoxin [32], paclitaxel [33], ligustilide [34]
Psoriasis and Antifungal	Psoralen [35], harmaline [36], baicalin [37], hesperidin [38]
Reduce UVB damage, Repair DNA injury, Anti-oxidative activity and skin whitening	Ferulic acid [39], cinnamic acid [40], usnic acid [41], menthol [42], pomegranate [43]

**Table 2:** Natural product transdermal delivery by the nanotechnology.

Compound	Technologies	Application	Publication Note
Bu- A	Liposome	Anti-inflammatory	2003 [102]
	Microemulsion	Improve solubility and stability	2019 [67]
Berberine	Nanocrystal nanosuspension embedded hydrogel-grafted fabric	Achieve sustained drug release	2014 [100]
Capsaicin	Microemulsion	Avoid gastrointestinal problems first-pass metabolism	2016 [103]
	Lipid nanoparticles	Increase permeation Lower skin irritation	2017 [83]
*Clinacanthus nutans Lindau*	Nanoemulsion	Improve Bioavailability	2016 [70]
Cumin essential oil	Nanoemulsion	prolonged systemic antioxidant	2015 [69]
Evodiae Fructus	Microemulsion Cataplasm	Improve Release performance	2015 [66]
Saussurea involucrata	Microemulsion	Enhance bioavailability	2012 [104]
Shortstalk Monkshood root	Microemulsion	Reduce side effects	2015 [105]
Triptolide	Nanoemulsion gels	Improve percutaneous amounts	2017 [64]
	Lipid nanoparticles	Enhanced skin permeation	2018 [73]
	Elastic liposomes	Remove Cremophor EL.	2011 [60]
Paeonol	Microemulsion gel	Enhance the skin permeability	2011 [65]
	Transethosomes	Improve stability, skin delivery pharmacokinetic efficiency	2017 [106]
	Ethosomes	Improve Bioavailability	2018 [107]
	Anti-VEGF Antibody-modified liposomes	Hypertrophic Scars	2019 [108]
Paeonol-menthol eutectic	Microemulsion	Enhanced skin permeation	2017 [109]
Paeonol and salidroside	Nanosphere-hydrogel system	For sequential delivery	2014 [88]
Resveratrol and 5-fluorouracil	Ultradeformable liposomes	Nonmelanoma skin cancer	2015 [55]
Resveratrol	Deformable liposomes	Avoid gastrointestinal problems and first-pass metabolism	2018 [110]
	Transfersomes	Maintain stability	2019 [54]
	Dendrimer	Improve bioavailability	2015 [111]
8-methoxypsoralen	Dendrimers	Enhance skin permeation	2012 [94]

**Table 3:** Natural products applied in cosmetics.

Scientific Name	Whitening	Moisturizing	Anti-Inflammation	Antimicrobial
*Aloe vera*	2008 [120]	2008 [120]	2008 [120]	2008 [120]
*Tribulus terrestris* L.	2015 [121]			2015 [121]
*Coix lacryma-jobi* L.	2017 [122]		2013 [123]	2017 [124]
*Atractylodes macrocephala Koidz*	2010 [125]		2007 [126]	2011 [127]
*Smilax glabra Roxb*	2010 [125]		2014 [128]	2013 [129]
*Paeonia lactiflora Pall*	2010 [125]		2019 [130]	2019 [130]
*Angelica dahurica Bentham et Hook.*	2006 [131]		2011 [132]	2011 [133]
**Nelumbo nucifera* Gaertn*	2013 [134]	2015 [135]	2015 [135]	2012 [136]
*Cyperus rotundus* L.	2015 [121]		2011 [137]	2006 [138]
*Glycyrrhiza glabra* L.	2003 [139]		2011 [140]	2019 [130]
*Eriobotrya japonica*			2011 [141]	
*Lithospermum erythrorhizon*	2015 [142]	2008 [143]	2007 [144]	1992 [145]
*Ligusticum chuanxiong Hort.*	2108 [146]		2011 [147]	
*Rheum officinale Baill*			2004 [148]	2007 [149]
*Lonicera japonica Thunb.*	2011 [150]		2011 [150]	2013 [151]
*Nardostachys chinensis Bat.*	2011 [152]		2014 [153]	2014 [153]
*Prunus armeniaca* L.	2011 [154]		2011 [154]	2009 [155]
*Typhonium giganteum Engl.*	2014 [156]		2014 [156]	
*Forsythia suspensa (Thunb.) Vahl*			2018 [157]	2018 [157]
*Angelica sinensis (Oliv.) Diels*	2011 [158]		2012 [159]	
*Zingiber officinale Rosc.*	2005 [160]		2005 [160]	2012 [161]
*Ajuga bracteosa Wall.*			2011 [162]	
*Rubus chingii Hu*	2009 [163]		2015 [164]	2012 [165]
*Cinnamomum aromaticum Nees*	2013 [166]		2012 [167]	2017 [168]
*Lythrum salicaria* L.		2011 [169]	2007 [170]	2007 [170]
*Plantago asiatica* L.	2019 [171]		2019 [171]	2008 [172]
*Salvia miltiorrhiza Bunge*	2015 [173]		2018 [174]	2011 [175]
*silkworm cocoon*		2019 [176]	2019 [176]	
*Quisqualis indica* L.	2008 [177]		2011 [178]	2008 [179]
*Ageratum conyzoides* L.			2005 [180]	2004 [181]
*Poria cocos (Schw.) Wolf.*	2017 [182]		2009 [183]	
*Scutellaria baicalensis Georgi*	2017 [184]		2006 [185]	2011 [186]
*Saposhnikovia divaricata (Turcz.) Schischk.*			2007 [187]	
*Talinum triangulare Willd.*	2013 [188]		2013 [188]	
*Bletilla striata (Thunb.) Reichb. f.*	2013 [189]	2015 [190]	2015 [191]	2013 [192]
*Leonurus japonicus Houtt.*	2018 [193]		2018 [194]	2013 [195]
*Evodia rutaecarpa (Juss.) Benth.*			2007 [196]	
*Ganoderma lucidum*	2008 [197]	2016 [198]	1993 [199]	

## Abbreviations

RSV	resveratrol
HLB	hydrophilic-lipophilic balance
CPP	critical packing parameter
TPL	triptolide
Bu-A	bulleyaconitine A
VCR	Vincristine
PUVA	psoralens and long-wavelength ultraviolet radiation
8-MOP	8-methoxypsoralen
